# Interplay of eco-friendly factors and islamic religiosity towards recycled package products: A cross-cultural study

**DOI:** 10.3389/fpsyg.2022.840711

**Published:** 2022-09-29

**Authors:** Qingyu Zhang, Mudassir Husnain, Muhammad Usman, Muhammad Waheed Akhtar, Saqib Ali, Mussadiq Ali Khan, Qamar Abbas, Riffat Ismail, Tayyab Rehman, Muhammad Akram

**Affiliations:** ^1^Research Institute of Business Analytics and SCM, College of Management, Shenzhen University, Shenzhen, China; ^2^Division of Management and Administrative Sciences, UE Business School, University of Education, Lahore, Pakistan; ^3^Department of Management Sciences, COMSATS University Islamabad, Sahiwal, Pakistan; ^4^Faculty of Economics and Business, Universiti Malaysia Sarawak, Sarawak, Malaysia; ^5^School of Management, Xian Jiaotong University, Xi'an, China; ^6^Lahore Business School, University of Lahore, Sargodha, Pakistan

**Keywords:** products with recycled packaging, pro-environmental factors, religiosity, theory of planned behaviour, cross cultural

## Abstract

Climate change has increasingly been recognised and associated with consumer behaviour: Practitioners are developing their strategies to reduce environmental degradation while increasing the management of sustainable consumption; it needs to better understand consumer attitudes and eco-friendly factors about the issue. Therefore, the current study focused to understand the effects of pro-environmental factors on individuals’ environmental attitudes (purchase behaviour towards products with recycled packaging) through the lens of theory of planned behaviour in a cross-cultural setting. Moreover, present research focuses on the moderating role that religiosity plays in causal pathways between certain determinants (attitude, subjective norms, and perceived behavioural control) and intentions in this context. A multi-wave time-lagged research design was employed in this study, and university students from two developing countries were surveyed (*N* = 324, 266). The findings revealed pronounced similarities between the two examined countries. Overwhelmingly, pro-environmental factors examined (environmental values, environmental knowledge, and environmental concern) were found to be positively related to attitude formation. Further results showed that attitude and subjective norms are significant predictors of the intention to purchase products with recycled packaging. Moreover, with the exception of perceived behavioural control, religiosity moderates the relationships between all the determinants of TPB and intention to purchase recycled packaged products. Present study offers insightful implications to management of these emerging and/or similar cultural markets regarding customer value for green products. Using TPB, present study broadened and deepen extant stream of literature on consumption of recycled packaged products in two highly emerging markets; Pakistan and Malaysia.

## Introduction

During recent times, rapid industrialization, unsustainable production and increasing pollution have depleted the ozone layer disproportionately. In their common race to develop innovative products, companies are using unsustainable means of production, including in their packaging of products with keeping view of public health (Despe et al., 2019). Packaging is thought to be a source of communication that affects brand popularity (Sheth, 2011), selection and purchase decisions (Han and Yoon, 2015). Although packaging provides a number of benefits, such as product safety, travel efficiency, and brand identity (Webb et al., 2017), it introduces serious environmental hazards. Annually, European countries produce approximately 163 kg of packaging waste per person, and the USA produces 218 kg per person (Eurostat, 2020). The situation is no different in developing countries, and this is reflected through the increasing pollution rates. Production and packaging not only generate enormous amounts of waste but also release emissions that contribute to global warming. Therefore, a reduction in packaging is best defined by its impact on waste; the extent to which such decisions can contribute to environmental sustainability and preservation (Guillard et al., 2018; Kautish et al., 2021). Despite its relevance, this issue has been largely overlooked and significantly lags behind in previous research (Guillard et al., 2018). From this perspective research on ecological packaging is scarce, while plethora of studies suggested that ecological factors play a substantial role in shaping environmental attitudes to scale individual buying behaviour (Prakash et al., 2019). Thus, there is a need to examine the pro-environmental consumer factors that affect attitudes, buying intentions and behaviours towards products with recycled packaging, which is the aim of the current study.

Furthermore, Davari et al. (2017) developed a model showing that religions are likely to encourage the values of humanity by promoting altruism, inducing believers to be in harmony with the environment. Early work attempted to establish a link between attitude and green purchase behaviour (Ali et al., 2019b), while studies, i.e., (Hameed et al., 2019a) and (Xu et al., 2020) found insignificant relationship of attitude with pro-environmental. In the same lines, studies such as (Ham et al., 2015) and (Hameed et al., 2019a) found significant relationship of subjective norm with green behaviour in Europe and Pakistan, but found insignificant in the studies of Ali et al. (2019b) and Si et al. (2019). Moreover, in case of green IT, PBC has also been considered as significant factor of green consumption (Dezdar, 2017) in Malaysia but recently found contradiction with the recent study of Zhang et al. (2018). The results of these studies were frequently inconclusive and sometimes contradictory thus calls attention to incorporate a moderating variable for such week relationships. Present study focuses on two different cultures, i.e., Pakistan and Malaysia. Although Islam is a religion practiced in both countries, a significant percentage of Malaysians practice other religions as well. Religion of Islam is practiced in the same way in both countries, these countries exhibit different consumption patterns (Saeed and Azmi, 2018). Thus, comparing these two countries may provide new insights into the concepts explored in the consumption and green marketing literature.

Considering the environmental concerns and social behaviours of consumers, significant environmental- and social norm-related predictors (environmental concern, knowledge, and value) were integrated into the theory of planned behaviour (TPB), which has been extensively applied in marketing research studies to understand pro-environmental consumer behaviour (Onel and Mukherjee, 2015a; Park and Lin, 2020). Despite the existence of evidence related to pro-environmental behaviours in the green marketing literature, few studies have examined how value and knowledge subsequently affect intentions and actual behaviour (Trang et al., 2019; Khan et al., 2021). The TBP is considered a useful perspective to predict consumers’ intentions and behaviour related to the environment (Chen and Tung, 2014). The dynamic nature of the TPB model provides an opportunity to incorporate additional constructs in the determination of consumers’ attitudes towards purchasing green packaging products. Therefore, the proposed study employs the fundamental constructs of the theory of planned behaviour with additional components such as religiosity.

Previous studies on green consumption have mostly focused on developed countries (Mufidah et al., 2018) and have largely ignored developing countries. Similar to other nations, Pakistan and Malaysia suffer from serious environmental and water pollution (Khan et al., 2016; Suki, 2016). In particular, Pakistan produces 50,438 tons of solid waste per day; 67% of this waste is organic, 5% is paper, and approximately 18% is plastic (Gill, 2018). Likewise, in Malaysia, only 15% of plastic waste is recycled properly (Bedi, 2018). According to Hofstede Insights (Insights, 2018), Pakistan and Malaysia slightly differ in terms of their cultural dimensions, i.e., individualism, power distance, and uncertainty avoidance. Integrating cross-cultural insights into pro-environmental psychology, this study may make a unique contribution to the pro-environmental behaviour literature, which comprises few studies currently (Tam and Chan, 2017). Moreover, despite the existence of several studies that mainly focus on determining pro-environmental predictors, little attention has been given to the role of cultural variations in predicting pro-environmental behaviours (Mintz and Kurman, 2020). Considering the universal nature of environmental problems, the development of a worldwide solution to address cross-cultural variations is essential (Morren and Grinstein, 2016). Based on aforementioned discussion, the study proposed the following research objectives:

- To analyse the effects of pro-environmental factors on consumer attitude towards consumption of recycled packaged products;
- To analyse the effects of predictor’s of TPB (environmental attitude, perceived behavioural control, subjective norms) on consumer intentions to buy recycled packaged products.
- To examine the moderating role of religiosity in the relationship between predictor’s of TPB and intention to buy recycled packaged products?
- To substantiate the effect of intention to buy recycled packaged products on consumer purchase behaviour and to assess whether such underlying mechanism contains cultural variations.

## Theoretical background and hypotheses

The following subsections provide a discussion of this study’s hypothesis development based on overarching theory.

### Theory of planned behaviour

The theory of planned behaviour is often used to explain the green consumption behaviour of consumers (Khan et al., 2019; Hameed et al., 2019b). Researchers have suggested that the TPB can be broadened by involving external variables that comprehensively explain phenomena (Ajzen, 1991; Perugini and Bagozzi, 2001). To strengthen the underpinning of the theory involving products with recycled packaging, this study integrates significant predictors (environmental concern, knowledge, and value) related to antecedents of attitude (Onel and Mukherjee, 2015b). Moreover, recent studies have observed the significant impact of religiosity on ecological behaviour (Ghazali et al., 2018) because preservations of environment and natural resources are found in guidance book of every religion (Ali et al., 2019a).

The TBP further suggests that individual behaviour significantly relies on attitudes and the social influence of society. These behavioural beliefs stimulate consumption behaviour towards eco-friendly products. Religion is an important construct linked with attitude, and it determines consumer intentions and behaviour. Therefore, it would be interesting to know whether such relationships are contingent on consumer religiosity (Cyril De Run et al., 2010).

### Environmental value

Environmental value (EV) has been defined as values that are actions oriented inclined towards the environment (Sony and Ferguson, 2017) and considered as determinants of individual’s attitudes and behaviours. Earlier literature primarily focused on three distinct aspects of individual’s environmental beliefs, i.e.; values, attitudes, and concerns; where Values has been used to define as ‘internal standards’ that exceeds in certain circumstances, and further assist to formulate certain attitudes and behaviours (Sony and Ferguson, 2017). Values initially affect a person’s attitude, which subsequently influences his or her behaviour; this pattern is called the value-attitude-behaviour hierarchy (Homer and Kahle, 1988; Sihombing, 2007).

Altruistic values and egoistic values influence environmental concerns (Schultz and Zelezny, 1998). Counterintuitively, such perspective seems more influential to generate emotional appeals and facilitate marketers to tailor better offerings for green products. So, the environmental value is only created if the person has positive attitude with the environment. Furthermore, this suggests that people with stronger environmental values have positive attitudes towards eco-friendly products (Suki, 2016). Therefore, a consumer who values the man-nature orientation will exhibit an attitude that supports green product consumption. In the light of the TPB framework and the above literature, we proposed the following hypothesis:

*H1(a)*: Environmental values have a positive effect on an individual’s attitude towards products with recycled packaging.

### Environmental knowledge

The environmental knowledge (EK) has been defined as individual’s ‘cognitive ability’ for comprehension of the environmental sustainability issues related to soil and water pollution, waste generation and recycling problems, and further their impact on physical environment specifically on the society at large (Saeed and Azmi, 2018). Earlier literature has primarily emphasised that environmental knowledge [ENK] is an important predictor of green consumerism (Wu et al., 2017). Researchers have argued that ENK directly affects consumers’ attitudes and purchase intentions towards ecological products; thus, higher levels of perceived environmental knowledge lead to increased purchase behaviour related to eco-friendly products (Yadav and Pathak, 2016).

Cerri et al. (2018) argued that ecologically conscious individuals are more sensitive to the environment than others; consequently, ENK influences an individual’s purchase intentions towards green products. Therefore, it is plausible that perceived environmental knowledge could be linked with attitude and intentions for the consumptions of the green products. Considering the above discussion, this study seeks to analyse the fundamental association between environmental knowledge and attitudes towards eco-friendly products. We proposed the following hypothesis:

*H1(b)*: Environmental knowledge has a positive effect on individuals’ attitudes towards products with recycled packaging.

### Environmental concern

Generally, environmental concerns (EC) influence consumers’ intentions to resolve environmental issues (Prakash and Pathak, 2017). Furthermore, they improve consumers’ sense of responsibility and passion regarding the protection of the environment at the individual level (Dagher and Itani, 2014). Hence, the consumer’s individual level environmental concern provoke the more alertness at the personal level that give way to the collective orientation towards pertaining environmental issues and the readiness to resolve them, ranging from waste recycling behaviour (Zhao et al., 2014) to green buying behaviour (Paul et al., 2016).

Previous studies have emphasized that environmental concerns are significantly related to attitudes towards eco-friendly packaging products that strengthen behaviours related to this issue (Yadav and Pathak, 2016). In addition, they are an efficient driver of environmentally friendly behaviours (Pagiaslis and Krontalis, 2014; Kautish et al., 2021). Therefore, environmental concerns can be viewed as a critical factor that affects the attitudes of customers towards eco-friendly packaging products. Thus, we proposed the following hypothesis:

*H1(c)*: Environmental concern has a positive effect on individuals’ attitudes towards products with recycled packaging.

### Attitude towards recycled products (A)

In the sphere of marketing, attitude is a process that guides consumers’ decisions regarding whether they will buy a specific product (Bhuian et al., 2018). Consumers’ attitudes towards intentions and purchasing decisions involving green products represents their awareness of environmental sustainability, which is different from their general environmental attitudes (Jaiswal and Singh, 2018). Several scholars have linked consumers’ awareness of environmental sustainability with green purchase intentions (Lai and Cheng, 2016) and purchase behaviour (Yadav and Pathak, 2016). Previous studies suggest that individuals with strong green attitudes are more inclined to purchase eco-friendly products (Joshi and Rahman, 2015). Therefore, if a consumer’s attitude towards a product is positive, his or her purchase intentions will be affected. Hence, consumers’ positive attitudes towards green products stimulate their readiness to purchase them (Cheah and Phau, 2011; Khan et al., 2021). Therefore, these positive attitudes are considered significant factors in stimulating green purchase intentions. Thus, we proposed the following hypothesis:

*H2*: Consumer attitude has a positive effect on consumer purchase intentions towards products with recycled packaging.

### Subjective norms

Subjective norms (SN) refer to the perceived social pressure placed on individuals to behave in a certain way due to the influence of their families, relatives, and close friends (Ajzen, 1991). Subjective norms have been found to be a significant construct in pro-environmental consumer behaviour (Yadav and Pathak, 2016). Herbes et al. (2018) found that the influence of subjective norms is culturally specific, and collective societies are more inclined to behave according to the influence of relevant reference groups. Hence, according to the consumer behaviour literature, an individual under the influence of subjective norms behaves in a positive way to create a positive social image among his peers, friends and family (Kumar et al., 2017). Consequently, subjective norms play an influential role in predicting consumers’ intentions to purchase specific products (Noble et al., 2009).

Furthermore, subjective norms have a significant relationship with attitudes and purchase intentions towards green products (Kim and Karpova, 2010), and are linked with consumer intentions (Paul et al., 2016) towards products with recycled packaging. Thus, based on the assumption of TPB, while purchasing recycled packaging products, consumers are influenced by opinion of the surroundings influential group of people. So, the above discussed literature proposed the following hypothesis:

*H3*: Subjective norms have a positive effect on consumer purchase intentions towards products with recycled packaging.

### Perceived behaviour control

Perceived behaviour control (PBC) refers to how individuals’ opinions influence certain behaviours (Ajzen, 2002). Yeon Kim and Chung (2011) found that PBC is associated with green purchase intentions. Hassan et al. (2016) stated, if the significant others are not inclined towards purchase of a specific product it will pose a psychological barrier for the customer to purchase it and ultimately negatively effect on individuals PBC. Therefore, we proposed that a high level of PBC will positively affect consumers’ green purchase intentions. Based on the TPB and the literature discussed above, the following hypothesis is formulated:

*H4*: Perceived behaviour control (PBC) has a positive effect on consumer purchase intentions towards products with recycled packaging.

### Behavioural intention

Behavioural intentions (BI) represent the readiness of an individual to engage in certain behaviour. A high level of intention towards a specific behaviour is likely to stimulate that behaviour (Ajzen, 1991; Husnain and Toor, 2017). In consumer psychology, behavioural intentions are a key factor in predicting consumers’ actual behaviour. Hence, incorporating green purchase intentions into pro-environmental studies is an important way to gauge actual eco-friendly behaviour (Yadav and Pathak, 2016; Khan et al., 2021). Kim et al. (2020) argued that intentions are greatly linked with attitudes to perform in a particular manner. So, the dedicated attitude will enhance the intentions of the consumers to make an actual purchase decision related to the behaviour.

Similarly, green products create environmental awareness among consumers, as purchasing is an important element of behavioural changes. Therefore, the literature reveals that purchase intentions are an essential element of determining behaviour (Lai and Cheng, 2016). Therefore, we proposed the following hypothesis:

*H5*: Purchase intentions have a positive effect on purchase behaviour for recycled packaging products.

### Islamic religiosity (R)

Since last few decades, religiosity as a research area has been extensively studied in western context. Research studies appeared in this domain were mainly referred and considered to Christianity faith (Raggiotto et al., 2018; Mortimer et al., 2020; Wang et al., 2020a). In literature, religiosity is differentiated from religion and can be defined as pattern of living that reflect in attitude, knowledge and values of the societal members (Akhtar et al., 2020b; Tsoraya et al., 2022). In turn, such cognitive beliefs and mannerism forms behaviours and practices of individuals and society (Samad et al., 2021; Tsoraya et al., 2022). Consequently, individual high on Islamic religiosity can dampen to low rate of deviant behaviour such as recycled packaged goods consumption. Considering the undeniable importance of Islamic religiosity in the context of pro-environmental behaviour, we examined the role of Islamic religiosity as a moderator in the relationship between the TPB constructs and purchase intention. In this research, we viewed Islamic religiosity as a moderator for the following reasons. First, the idea of viewing Islamic religiosity as a moderator between the TPB constructs and the intention to buy recycled products was taken from contingency theory, which proposes that the relationship between two variables is dependent or contingent on a third variable. Therefore, the involvement of Islamic religiosity as a moderator in the examined relationship leads to a better understanding of the related phenomena and helps avoid misleading ideas regarding contingency relationships among the TPB constructs. Similar to the way that contingency theory (Venkatraman, 1989) contributes to the understanding of inconsistent attitudes, SN and PBC contribute to the understanding of intentions. Second, a recent studies on pro-environmental behaviour (Bhuian et al., 2018; Soomro, 2019) suggested that the moderating role of Islamic religiosity should be considered in the context of the relationships between the antecedents of TPB and consumers’ purchase intentions towards eco-friendly products. Moreover, researchers in the fields of marketing and consumer behaviour agree that the concept of Islamic religiosity is significant in thoroughly explicating pro-environmental consumer behaviour (Setyawati et al., 2020; Nickerson et al., 2022). However, very little research has been specifically concerned with demonstrating the role of religiosity in non-western contexts, i.e., Malaysia and Pakistan (Wang et al., 2020a). Finally, in the context of green IT, PBC has been viewed as a significant factor influencing green consumption (Dezdar, 2017) in Malaysia.

The studies that focus on investigating the correlation between religion and the intention to buy have validated the notion that religion significantly affects an individual’s preferences (Graafland, 2017). However, the extent to which religion affects an individual varies from person to person. Ultimately, it is not only a person’s religion but also the passion and intensity with which he or she holds his or her religious beliefs and affiliation (religiosity) that matter in the context of his or her intention to buy. Therefore, religiosity as a whole affects an individual’s thinking, lifestyle, and buying decisions. Similarly, Stern (2000) suggested that consumers with a high level of religiosity exhibit strong pro-environmental attitudes that consequently affect their intentions to buy.

**Figure 1:**
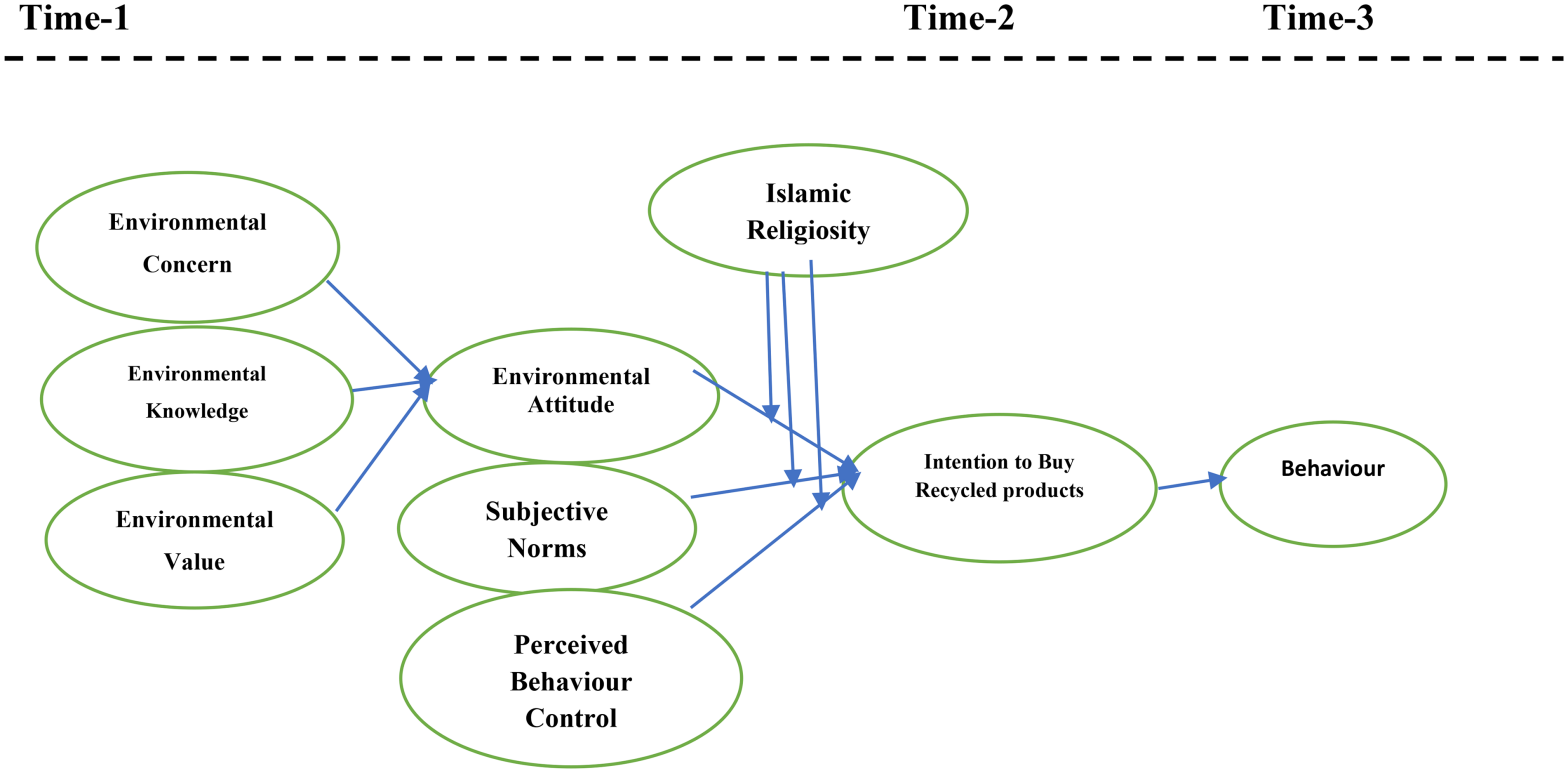
Theoretical framework of the study.

Islamic Religiosity could be a significant construct to analyse in the settings of Islamic societies such as Pakistan and Malaysia, particularly in the context of consumer attitudes towards products with recycled packaging. Figure 1 shows the theoretical framework of our study. Based on the TPB framework and the literature discussed above, the following hypotheses were proposed:

*H6*: The Islamic religiosity of Muslim Consumers moderates the positive effect of environmental attitude (EA) on their intention to buy (IB) such that it will be stronger for a more devout religious person than a less devout one.

*H7*: The Islamic religiosity of Muslim Consumers moderates the positive effect of subjective norms (SN) on their intention to buy (IB) such that it will be stronger for a more devout religious person than a less devout one.

*H8*: The Islamic religiosity of Muslim Consumers moderates the positive effect of perceived behavioural control (PBC) on their intention to buy (IB) such that it will be stronger for a more devout religious person than a less devout one.

## Research methodology

The following subsections discuss the design, population, sample, procedure and measures used in this study.

### Research design

A time-lagged approach was used to collect the data at three points in time. Temporal research designs view the causes as preceding the effects in time; this makes temporal precedence a necessary provision for causation in a model (Cook et al., 1979). It is very convenient in the social sciences to determine time lags and researchers opts such design to minimize social desirability bias (Cole and Maxwell, 2003; Husnain et al., 2021, 2022). The constructs of pro-environmental attitudes, subjective norms and perceived behaviour control were measured at Time 1, those of religiosity and the intention to buy recycled packaging products were measured at Time 2 (2 weeks after Time 1), and that of consumer purchase behaviour was measured at Time 3 (2 weeks after Time 2). The data were collected between April and June 2019 in three rounds. For time 1, data on all the independent and moderating variables were collected during the first 3 weeks of April; 2 weeks after Time 1, data were collected from the same respondents for the mediating variables; and 2 weeks after Time 2, data were collected for all the dependent variables. A non-random purposive sampling technique was used. The units of analysis examined were students who are currently enrolled in different semesters of under graduation and post-graduation programs, i.e., environmental science, in different universities of Pakistan and Malaysia. The foremost reason for employing this students sample was that young people have high need for uniqueness with more inclination towards adoption of new ideas than others (Ottman et al., 2006) and have more concern on recyclability issues (Nuojua et al., 2022). The respondents that were recruited were offered modest compensation to ensure their participation in the study. A personally administered questionnaire was devised to collect the statistical data required for the study. For the Pakistani sample, initially, 500 questionnaires were distributed at time 1, and 418 of these were received back; 372 questionnaires were returned at time 2, and 340 were returned at time 3. Of these, 324 were found to be complete in all aspects and useable for the study. Likewise, in Malaysia, 300 questionnaires were given to the respondents at time 1, and 290 were returned; 286 questionnaires were returned at time 2, and 278 were returned at time 3. Of these, 266 were considered useable. A total of 590 questionnaires formed the sample used for the study.

### Measure

Present study has employed combination of several scales that were adapted and modified with respect to cross-cultural context of the research (See Appendix 1). Such adaption to construct items is desirable to reinforce the respondent understanding, whose intrinsic meanings can manifest differently across settings (Rudran and Kumar, 2017). Meanwhile adapted constructs scale and items were evaluated by known expert researchers to ensure face validity. All the constructs were measured on a 5-point Likert scale; 1 denoted “strongly disagree” and 5 denoted “strongly agree.” Environmental concern was measured using a 4-item scale adapted from the work of Sivek (2002). Environmental knowledge was measured with an 8-item scale proposed by Haron et al. (2005). Environmental values were measured with 4 items adopted from the work of De Groot et al. (2007). A measurement scale adapted from the work of Huang et al. (2004), which contained 5 items, was employed to measure attitudes. Subjective norms were measured using 4 items adopted from the work of Ajzen (1991). To measure perceived behaviour control, a 3-item scale taken from the study of Martinho et al. (2015) was used. To conceptualize religiosity, a 13-item scale was adopted from the work of Eid and El-Gohary (2015). The behavioural intention to buy recycled packaging products was measured using 5 items adapted from the work of Zeithaml et al. (1996). Actual environmental consumer behaviour was measured using 4 items adapted from the work of Schlegelmilch et al. (1996).

## Analysis and results

The following sections explain the data analysis and results.

### Demographics

The sample of this study was composed of 453 respondents from Pakistan (*N* = 310) and Malaysia (*N* = 143). The demographic details of the respondents are shared in Table 1. To mitigate potential systematic errors, gender and education were included in the structural model as control variables.

**Table 1:** Demographics of the respondents.

Characteristics	Total Sample *N* = 453	Pakistan *N* = 310	Malaysia *N* = 143
Gender			
Male	291	161	130
Female	162	98	64
Age			
18–22 Years	129	89	40
23–26 Years	174	83	91
27–30 Years	95	49	46
> 30 Years	55	26	29
Income			
1–20,000 PKR	127	58	69
20,001–40,000 PKR	189	72	117
40,001–60,000 PKR	79	31	48
> 60,000 PKR	58	16	42
Qualifications			
Undergraduate	202	121	81
Master	162	84	78
M.Phil. & above	89	38	51

Income in Malaysian currency is converted to Pak Rs.

### Assessment of the measures and common method bias

To verify the constructs’ independence and to validate the discriminant validity of the study variables, a confirmatory factor analysis was performed. Appendix 1 shows that the significant factor loadings >0.50 (Hair et al., 2014) and that the magnitude of the average variance extracted (AVE) > 0.5 as recommended by Bagozzi and Yi (2012). While this confirmatory factor analysis (CFA) was performed, to obtain better model fit indices, several items, i.e., one item regarding environmental concern (EC), two items regarding subjective norms (SN), one item regarding religiosity (R), and three items regarding behavioural intentions (BI), were excluded due to poor factor loadings. The discriminant validity of all the constructs in Table 2 shows that the square root of the correlations was less than the AVE estimates (Fornell and Larcker, 1981). Table 3 shows the results regarding both countries and demonstrates that the hypothesized model has a good fit (χ^2^/Df = 1.72, *p* < 0.01; root mean square residual [RMR] = 0.046; goodness fit index [GFI] = 0.91; comparative fit index [CFI] = 0.88; and root mean square error of approximation [RMSEA] = 0.051).

**Table 2:** Results regarding discriminant validity: aggregate.

	1	2	3	4	5	6	7	8	9
1. INT(T2)	(0.741)								
2. ENC(T1)	0.258	(0.863)							
3. ENK(T1)	0.531	0.356	(0.779)						
4. ENV(T1)	0.176	0.184	0.386	(0.680)					
5. ATT(T1)	0.394	0.298	0.415	0.209	(0.702)				
6. SN(T1)	0.278	0.368	0.298	0.189	0.388	(0.892)			
7.PBC(T1)	0.179	0.207	0.190	0.302	0.201	0.289	(0.731)		
8.REL(T2)	0.278	0.498	0.283	0.491	0.245	0.489	0.426	(0.720)	
9.BEH(T3)	0.173	0.287	0.389	0.315	0.278	0.308	0.288	0.387	(0.825)

N = 453; T = Time; the values in brackets are the square roots of the (AVE).

**Table 3:** Results of the model comparison (Pakistan and Malaysia).

Model	χ^2^/Df	RMR	GFI	CFI	RMSEA
Hypothesized model	1.57/1.73	0.04/0.05	0.94/0.89	0.88/0.85	0.04/0.05
Three-factor model^a^	14.9/9.45	0.14/0.19	0.53/0.51	0.59/0.68	0.02/0.03
Three-factor model^b^	11.5/9.02	0.16/0.23	0.55/0.68	0.61/0.50	0.12/0.14
Three-factor model^c^	8.01/7.53	0.15/0.21	0.65/0.60	0.54/0.62	0.39/0.82
Three-factor model^d^	24.1/15.6	0.23/0.22	0.45/0.52	0.61/0.57	0.13/0.14
Single-factor model	34.2/28.5	0.13/0.06	0.22/0.31	0.33/0.39	0.37/0.16

Data from Pakistan and Malaysia were examined in this analysis; ^a^attitude, subjective norms, perceived behavioural control, and religiosity were combined in a model; ^b^attitude, subjective norms, perceived behavioural control, and intentions were combined in a model; ^c^religiosity and intentions were combined in a model; ^d^intentions and behaviour were combined in a model; N = 453; RMR, Root mean square residual; GFI, Goodness of fit index; CFI, Comparative fit index; and RMSEA, Root mean square error of approximation.

In this study, several diagnostic analyses and procedures were conducted to control for common method bias (CMB). To control potential effects of self-reporting bias, survey contains neutrally worded questions to ensure answer options are not leading (Podsakoff et al., 2003; Brannick et al., 2010). To reduce the potential for CMB, the present study used a time-lagged design.

Two statistical techniques, Harman (1967) and marker variable were used to examine the potential effects of CMB. Applying Harman’s one factor test, all the factors were constrained into a single factor and results showed that sole factor can only explain 23 and 29% of total variance for Pakistani and Malaysian sample, respectively, indicating that there is no problem of CMB. Additionally marker variable approach, in which common variance was calculated by getting square root of each path to common factor. Results indicate that estimated common variance was below 50% for both samples (35% in the Pakistan, 28% in the Malaysia) further providing assurance that there is no issue of common method bias in this study.

The discriminant validity of the variables was established by contrasting the hypothesized model with several three-factor models and a single-factor model. The comparison results corresponding to the models (Table 3) of both countries, i.e., Pakistan and Malaysia indicated that the additional models examined, including a three-factor model combining attitude, subjective norms, perceived behaviour control and religiosity [χ^2^/Df = 14.95; RMR = 0.14; GFI = 0.53; CFI = 0.59; and RMSEA = 0.02], a three-factor model combining intention towards recycled products and behaviour [χ^2^/Df = 24.15; RMR = 0.23; GFI = 0.45; CFI = 0.61; and RMSEA = 0.13] and a single-factor model [χ^2^/Df = 34.23; RMR = 0.13; GFI = 0.22; CFI = 0.33; and RMSEA = 0.37], did not fit the data as well as the hypothesized model. Thus, the discriminant validity of the hypothesized variables was supported.

### Analysis of the structural models and hypotheses testing

The present study uses structural equation modelling technique to test the hypotheses using maximum likelihood (ML) estimation method. This method is capable to obtain estimation for parameters form multivariate normal distribution as well as non-normally distributed data. For the structural model, the model fit indices for the Pakistani data (χ^2^/Df = 1.830; RMSEA =0.047; CFI = 0.914; GFI = 0.902; TLI = 0.894; and AGFI = 0.79) and the Malaysian data (χ^2^/Df = 1.836; RMSEA = 0.051; CFI = 0.87; TLI = 0.76; GFI = 0.81; and AGFI = 0.90) were examined. The RMSEA is considered one of the most informative criteria for examining how well a hypothesized model fits observed data (Bagozzi and Yi, 2012); hence, a good error of approximation indicates a good fit. Thus, the results indicate that the data fit the hypothesized model well.

Table 4 shows the explanatory power (*R*^2^) of the model’s estimates of the endogenous constructs. For the Pakistani sample, it is evident that the proposed model exhibits a high level of explanatory power for environmental attitude (*R*^2^ = 0.41), while it exhibits a relatively low level of explanatory power for behavioural intentions (*R*^2^ = 0.26) and buying behaviour towards recycled products (*R*^2^ = 0.62). Similarly, for the Malaysian sample, the proposed model shows a high level of explanatory power for environmental attitude (*R*^2^ = 0.39) and buying behaviour towards recycled products (*R*^2^ = 0.46) but a lower level of explanatory power for behavioural intentions (0.21). Hair et al. (2011) proposed that the *R*^2^ values of 0.75, 0.50, or 0.25 can be employed to indicate substantial, moderate, or weak explanatory power, respectively. Therefore, per these standards, one value corresponds to substantial explanatory power, five values to moderate explanatory power and two to weak explanatory power. Moreover, regarding the hypothesis tests for both samples, Table 4 shows the respective significance levels of the path coefficients.

**Table 4:** Results regarding the main effects and the moderated regression analyses.

Criterion	Predictors	Hypothesis	*R* ^2^	Pakistan path coefficient		*R* ^2^	Malaysia path coefficient
EN	ENV(T1)	H1(a)	0.41	0.17^*^		0.39	0.11^*^
Attitude(T1)	ENK(T1)	H1(b)		0.44^**^			0.26^*^
	ENC(T1)	H1(c)		0.31^*^			0.17^**^
Intentions(T)	Attitude(T1)	H2	0.26	0.59^**^		0.21	0.55^**^
	SN(T1)	H3		0.28^*^			0.22^*^
	PBC(T1)	H4		n.s.			n.s.
Behaviour(T3)	Intentions(T2)	H5	0.62	0.46^***^		0.46	0.39^*^
*Moderation* Predictors	Criterion	C.R	*R* ^2^	Estimate Pak	C.R	*R* ^2^	Estimate MY
ZINT	ZATT	4.62	0.42	0.13^*^	3.43	0.33	0.26^**^
	REL_ × _ATT	5.45(H6)		0.22^*^	4.73		0.14^*^
	REL_ × _SN	4.69(H7)		0.19^**^	4.26		0.10^*^
	ZSN	6.56		0.33^**^	5.70		0.45^**^
	ZREL	5.02		0.48^*^	6.33		0.25^*^
	ZPBC	1.03		n.s.	n.s		n.s
	REL_x_PBC	0.78(H8)		n.s.	n.s		n.s

N = 310 (Pak); 143(MY). EN = Environment; ENV = Environmental values; ENK = Environmental knowledge; ENC = Environmental concern; PBC = perceived behavioural control; SN=Subjective norms; and REL = Religiosity. Significance (two-tailed test):

*** p < 0.001;

** p < 0.01;

* p < 0.05.

First, considering the Pakistani sample, the findings with respect to environmental values, (*β* = 0.17 and *p* < 0.01), knowledge (*β* = 0.44 and *p* < 0.01), and concern (*β* = 0.31 and *p* < 0.05) provide good support for H1a, H1b and H1c, respectively. Environmental attitudes and subjective norms were found to have significant positive effects on intentions (*β* = 0.59 and *p* < 0.01; *β* = 0.28 and *p* < 0.05), confirming H2 and H3, while perceived behavioural control was found to be insignificant at *p* > 0.05 (H4 was not supported).

In the Malaysian sample, similar results were found in terms of confirming the predictors of environmental attitude as environmental values (*β* = 0.11 and *p* < 0.05), environmental knowledge (*β* = 0.26 and *p* < 0.01), and environmental concern (*β* = 0.17 and *p* < 0.01). Similarly, environmental attitude and subjective norms had significant positive effects on intentions (*β* = 0.55 and *p* < 0.01; *β* = 0.22 and *p* < 0.05), supporting H2 and H3. Similar to the Pakistani sample, perceived behavioural control did not have an effect on intentions; thus, H4 was not supported in the Malaysian sample.

### Testing moderation

A moderated regression analysis was used to test the moderating role of religiosity. To examine the moderating effects suggestion of Gaskin (2012b) was followed, the interaction term of each relationship was produced by multiplying the standardized value of the moderator, i.e., religiosity, with each predictor’s standardized value (environmental values, environmental knowledge, and environmental concern). As indicated in Table 4, the interaction between environmental attitude and religiosity (REL_x_ATT) was positively related to intentions in the Pakistani and Malaysian samples (*β* = 0.22^*^ and *p* < 0.05; *β* = 0.14^*^ and *p* < 0.05). These findings are in line with H6. Regarding H7, the interaction between social norms and religiosity (REL_x_SN) was found to be significant in both the Pakistani and Malaysian samples (*β* = 0.19^**^ and *p* < 0.01; *β* = 0.10^*^ and *p* < 0.05). Finally, as shown in Table 4, the interaction between perceived behavioural control and religiosity (REL_x_PBC) did not moderate the relationship between perceived behavioural control and intentions in either country’s sample (*β* = 0.08 and n.s; *β* = 0.01 and n.s). Hence, H8 was not supported.

**Figure 2:**
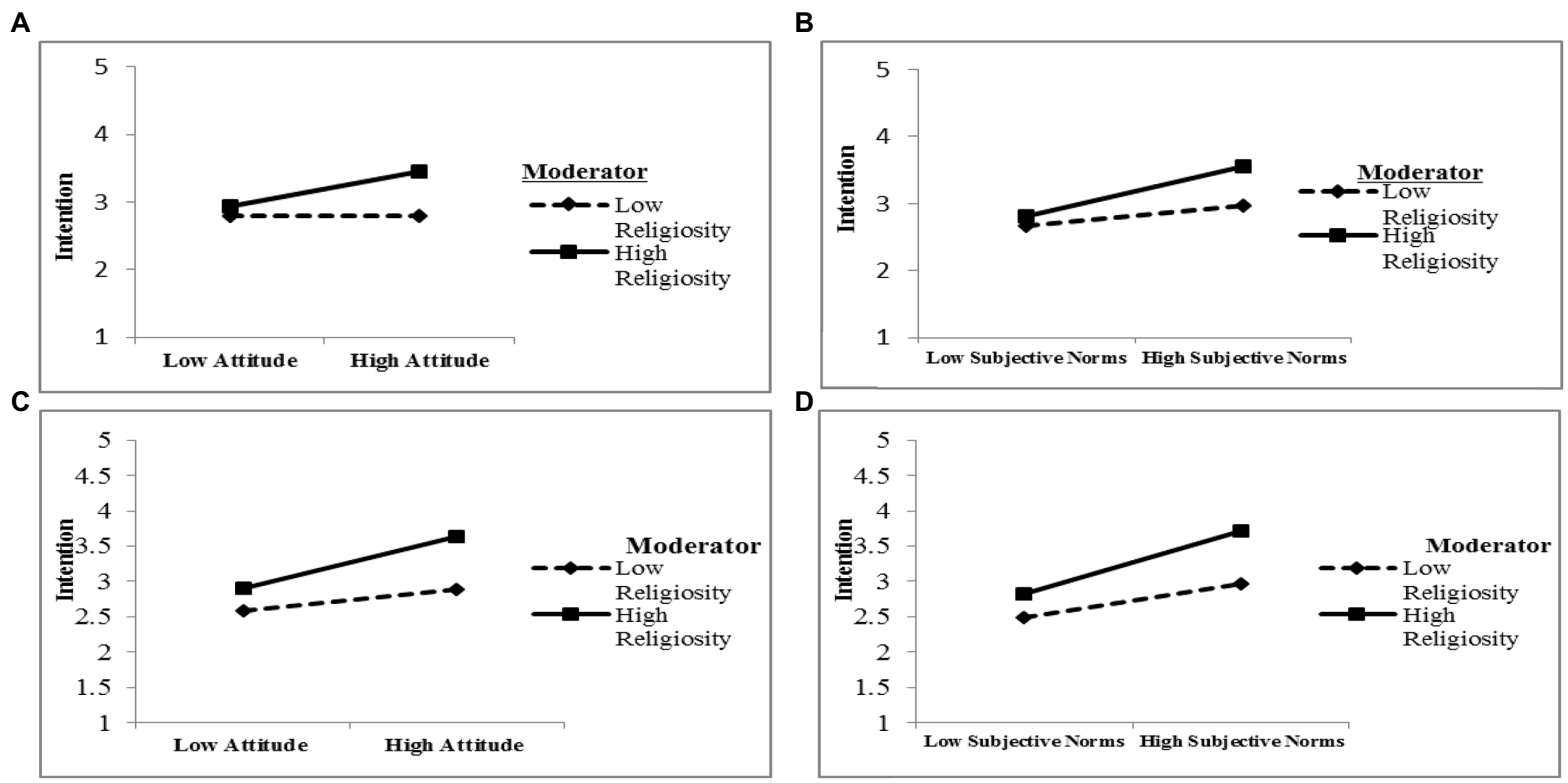
Moderator Interactions i.e. Religiosity x Attitude, Religiosity x SN, Religiosity x PBC.

Gaskin (2012a) suggestion was followed in interpreting the moderating effects. As Figures 2A,B show, it was concluded that the patterns of religiosity’s interactive effects were consistent with the prediction of H6; in particular, environmental attitude (shown on the horizontal axis) had a stronger positive effect on the intention to use recycled products (shown on the vertical axis) when the examined consumers showed high levels of religiosity (shown on the straight line) than when the consumers exhibited low levels of religiosity (shown on the dotted line). Thus, H6 was supported for both samples. Similarly, Figures 2C,D show that the pattern of interaction was consistent with the prediction of H7; that is, subjective norms (shown on the horizontal axis) had a stronger positive effect on the intention to use recycled products (shown on the vertical axis) when the consumers showed high levels of religiosity (shown on the straight line) than when they exhibited low levels of religiosity (shown on the dotted line). Hence, H7 was supported.

## Findings and discussion

The current study examined the Pro-environmental factors that affect individuals’ attitudes towards products with recycled packaging by examining the essential constructs of the TPB and the moderating role of religiosity in the cross-cultural contexts of Pakistan and Malaysia. The findings of this study strongly supported the applicability of the TPB to the field of ecological studies. Moreover, the findings supported the employment of additional pro-environmental factors (PEFs) as constructs in the TPB model, as these factors enhanced the predictive power of the proposed model, which examined environmental attitudes and behaviour towards products with recycled packaging.

The current study revealed that the exogenous determinants of environmental attitude, i.e., ENV, ENK, and ENC, significantly influence consumer attitudes, indicating that young people’s concerns about environmental issues related to their ecological choices influence their purchase decisions in both Pakistan and Malaysia. A close examination of the extant literature revealed that several studies that were conducted in Pakistan and Malaysia acknowledged the importance of ENV (Latif et al., 2012), ENK (Hasan et al., 2015; Ansari and Siddiqui, 2020) and ENC (Suki, 2016; Wang et al., 2020b) in affecting environmental attitudes and environmental behaviour. This may be because Malaysia and Pakistan are majority-Muslim countries, as it is evident that the green movement has expanded rapidly in Muslim-populated countries (Hasnah Hassan, 2014; Ali et al., 2019a).

The current study also found that subjective norms are a strong predictor of consumer purchase intentions involving recycled packaged goods in both countries, reflecting the strong moral values of the young consumers in these nations regarding the environment. Moreover, these findings are in line with past research showing that subjective norms affect pro-environmental consumer behaviour (Danish et al., 2019; Saleki et al., 2019; Yuriev et al., 2020). The probable reason for this result is that both Pakistan and Malaysia are collectivist societies. To avoid conflicts in decision-making, people prioritize others in a collectivist society (Ghazali et al., 2017; Ali et al., 2019a).

Thus, the current study was in line with past research showing that ENC stimulates eco-friendly behaviour and a sense of responsibility to behave in an eco-friendly way (Suki, 2016). Consistent with the past research of Martinho et al. (2015), ENK was found to be an important factor affecting the attitudes of individuals, which subsequently affect their behaviour towards recycled packaging products. Moreover, the findings showed that ENK is a weaker predictor of this behaviour than environmental values; thus, the importance of creating more awareness about environmental issues among youth to encourage societal development was demonstrated.

The results of the study revealed that the attitudes of the examined Pakistani and Malaysian student consumers were the most significant factors affecting their purchase intentions towards products with recycled packaging. These findings were consistent with previous studies showing that individuals with greener attitudes are more inclined to purchase eco-friendly or green products (Joshi and Rahman, 2015; Manosuthi et al., 2020). Therefore, these young consumers’ positive attitudes were a good indication of their motivation to purchase eco-friendly products.

The current study also identified subjective norms as a strong predictor of consumer’s purchase intentions regarding recycled packaged goods in both countries, reflecting the strong moral values of young consumers towards the environment. Moreover, these findings were in line with past research showing that subjective norms affect pro-environmental consumer behaviour (Yadav and Pathak, 2016; Ahmad et al., 2021). Additionally, the current study confirmed the findings of Han et al. (2018), namely, that subjective norms and their impacts on individual behaviours vary in the contexts of different cultures (Akhtar et al., 2020a). The findings reconfirmed that in collective cultures such as Pakistan and Malaysia, subjective norms have a considerable effect on purchase intentions.

The current study investigated the moderating role of religiosity in the relationships between the predictors of pro-environmental attitudes (environmental concern, environmental knowledge and environmental values), subjective norms and perceived behaviour control and the intention to buy products with recycled packaging. The results show that with an increase in religiosity, the relationships among pro-environmental attitudes, subjective norms and the intention to purchase green products become stronger in both samples. These findings are consistent with past research showing that religiosity moderates the relationship between environmental attitudes and intentions (Felix and Braunsberger, 2016; Radic et al., 2022). Additionally, the current study reflects that religiosity enhances the moral values of young consumers that favour environmental protection, which reflects the interplay between religiosity and subjective norms. These findings are consistent with the prior studies in this field (Taufique et al., 2017).

Interestingly results in present study reveal that religiosity did not moderate the relationship between perceived behaviour control (PBC) and the intention to purchase products with recycled packaging thus not supporting H3. The theory of planned behaviour relates PBC to the self-efficiency of an individual’s perception of how complicated and difficult it is to show certain behaviours (Ajzen, 2002). One possible reason might be cultural orientation of the studied countries, i.e., Pakistan and Malaysia, exhibit a high level of collectivism (Hofstede and Bond, 1984), meaning that individuals in these countries usually cannot make independent decisions because the prevailing joint family systems are characterized by central control held by the head of a family. Thus, consumption decisions are made by the leading members of a family. These findings are consistent with those of the previous studies (Jain et al., 2017) arguing that a positive attitude towards green products does not necessarily guarantee a purchase under the influence of PBC. Hence, PBC is not an influential factor in that influences consumer attitudes and behaviour, but it is certainly linked with significant beliefs that are viewed as key factors in decision making (Yazdanpanah and Forouzani, 2015).

Finally, the results of this study revealed that the intention to purchase recycled packaged products has a significant effect on purchase behaviour, reflecting the positive relationship between behavioural intentions and actual behaviour. Based on the tenets of the TPB, a high level of intention is likely to stimulate stronger behaviour in an individual (Ajzen, 1991). These findings are consistent with the related previous research (Abbey et al., 2015; Khor and Hazen, 2017).

## Implications of the study

This study made a significant contribution in terms of bridging the existing knowledge gap in the literature and providing practical implications for the development of consumers’ attitudes and purchase behaviours regarding recycled packaging products. Theoretically, the present study broadened the TPB framework by incorporating pro-environmental factors, i.e., (ENV), (ENK) and (ENC), and deepened the TPB framework by adding religiosity (Perugini and Bagozzi, 2001). This was the first study to broaden and deepen the TPB framework in cross-cultural settings with the intention of investigating pro-environmental behaviour. Numerous green marketing scholars around the world have argued that a lack of foreign market information is always a major concern in creating customer value for green products (Lee, 2017). The similar results presented herein offer insights to policy makers and marketers about the Muslim consumer market segment, who are estimated to consume $3.2 trillion in food and lifestyle products worldwide by 2024 (Salaam Gateway, 2020). However, this massive Muslim consumer market segment is largely under-researched (Ali, 2017). The Muslim consumer market segment is considered homogeneous because the Muslim lifestyle is guided by the scripture, namely, the “Quran” and “Sunnah” of Islam (Ali, 2017; Ali et al., 2019a). According to the Global Islamic Economy Report 2020, Malaysia ranks 1st in the Global Islamic Economy among the Muslim countries. Furthermore, according to the World Population Review (2020), Pakistan is the 2nd most populous Muslim country in the world, as it provides a home to 11% of the world’s Muslims. In addition, a recent Pew Report projected that by 2050, Muslims will make up 30 percent of the world’s population (Ali, 2017). These statistics have drawn the attention of policy makers towards the Muslim consumer segment. Thus, results similar to those of this cross-cultural study suggest that green marketers should formulate targeted strategies to enhance pro-environmental behaviours in Pakistan and Malaysia specifically, as well as in similar cultural settings.

Products with recycled packaging are projected to influence consumer’s health in broader way than ordinary packaged products being currently consumed. Consequently, health focus consumers might tend to inclined more towards ecological products (Birch et al., 2018). Findings of present study offer numerous implications for managers. First, as mentioned earlier, packaging material used for fast moving consumer goods (FMCGS) and groceries accounts for 75% of the total packaged products, thus considered main source of waste in solid form. Accordingly, waste management should emphasize the consumption of eco-friendly recycled packaged products to mitigate such mounting solid waste. Second, findings of present study confirmed the role of environmental attitudes in understanding young consumer response, i.e., Pakistan and Malaysia, for recycled packaged products that will assist managers to redesign the packaging. Also being religiously inclined consumers, practitioners are advised to put more efforts in their promotional strategies by communicating environmental benefits of ecological products *via* opinion leader’s, i.e., religious leaders. Pakistan and Malaysia are multicultural countries and account for 64% of the population under the age of 30 (UNDP, 2018); thus, they are ranked as one of the largest markets in terms of young consumers. Our analysis of a sample of young university graduates suggested that sustainable eco-friendly standards provide numerous benefits. Therefore, marketers should consistently attempt meet these individuals’ expectations regarding eco-friendly products with recycled packaging. The present study offers an opportunity for marketing professionals and managers to clearly understand their target population’s religious beliefs in terms of how they shape recycled product attitudes and ecological consumption patterns. The uniqueness of this study is that it showed that religiosity acts as a significant moderator of the relationship between pro-environmental attitudes and subjective norms. This validated the important role of religiosity in determining ecological behaviours. Therefore, marketers and managers can integrate religious factors into their marketing strategies to address consumers’ green behaviours and consumption patterns.

## Limitations and future research direction

Despite the numerous strengths of this study and the fact that we addressed a novel domain of inquiry through it, the current research is not free of limitations. The sample used comprised young university graduates, and young consumers are usually more aware and socially responsible individuals (Yadav and Pathak, 2016; Juan et al., 2020); this might mean that the results of the current research were biased and cannot be generalized to other contexts. The use of a sample of diverse demographic populations with distinct religious affiliations is recommended for future studies to generalize these findings.

Furthermore, this study took general religious beliefs as a moderator, but future studies could employ intrinsic or extrinsic religiosity (Mokhlis, 2009) as a moderator to broaden the examination of the research phenomena. Similarly, (Schirmer et al. (2018)) considered personality traits as potential moderators in understanding consumer behaviour. Therefore, future studies should integrate these factors as moderators in the TPB framework to measure individuals’ pro-environmental attitudes and green purchase intentions.

Additionally, this study focused on only recycled packaging, but did not address products. Therefore, future studies should focus on a variety of eco-friendly products. Furthermore, a time lag study was conducted, which limited our ability to establish causality; however, future studies can employ pure longitudinal designs to identify changes in the attitudes of individuals and the levels of their religiosity over time.

Overall, the current study is very timely and responds to the recent calls regarding environmental concerns and the establishment of green marketing constructs to identify the process by which consumers’ attitudes transform into intentions and then to actual buying behaviours. The current research provides multiple implications for marketing professionals and opens up several avenues for future researchers in the field of green marketing, as explained above.

## Data availability statement

The raw data supporting the conclusions of this article will be made available by the authors, without undue reservation.

## Ethics statement

The studies involving human participants were reviewed and approved by COMSATS University Islamabad (CUI), Sahiwal Campus constitutes Campus Ethics Approval Committee. The patients/participants provided their written informed consent to participate in this study.

## Author contributions

The idea of this research was suggested by MH who wrote the initial protocol of this study and QZ critically revised the important literature of the manuscript. MA further collect the data in revisions while MU, and SA performed some new statistical analysis as suggested by reviewers. MWA, RI, and TR maintained the respondents list at time2 and time3 during revisions and wrote the conclusion and implications. MAK re-run the analysis and interpretation of data, while QA revised discussion portion. All authors contributed to the article and approved the submitted version.

## Funding

This research was supported by Key Project of the National Social Science Foundation of China (21AGL014).

## Conflict of interest

The authors declare that the research was conducted in the absence of any commercial or financial relationships that could be construed as a potential conflict of interest.

## Publisher’s note

All claims expressed in this article are solely those of the authors and do not necessarily represent those of their affiliated organizations, or those of the publisher, the editors and the reviewers. Any product that may be evaluated in this article, or claim that may be made by its manufacturer, is not guaranteed or endorsed by the publisher.

## Figures and Tables

**Table:** 

**Constructs**	**Items**	**Factor Loading**		**CR**		**AVE**	
		**Pak**	**Mal**	**Pak**	**Mal**	**Pak**	**Mal**
**Environmental values**	EV1: “The balance of nature is very delicate and easily upset”	0.71	0.67	0.78		0.83	0.70		0.74
	EV2: “Humans must live in harmony with nature in order to survive”	0.73	0.75					
	EV3: “Mankind was created to rule over the rest of nature”	0.67	0.79					
	EV4: “Humans have the right to modify the natural environment to suit their needs”	0.82	0.63					
**Environmental Knowledge**	EK1: “Most smog in our big cities comes from industrial plants.”	0.68	0.69	0.69		0.85	0.72	0.67
	EK2:“Unacceptable levels of mercury have been found in seafood.”	0.78	0.89					
	EK3: “Diesel fuel generates less pollution than unleaded fuel.”	0.65	0.84					
	EK4: “Ecology assumes that man is an integral part of nature.”							
	EK5: “Aluminium takes longer to decompose than iron or steel.”							
	EK6: “Traditional plastic bags do not decompose in landfills.”	0.81	0.66					
	EK7: “Products do not use any electricity in sleep mode.”	0.69	0.58					
	EK8: “Most of the water in the country is used in agriculture.”	0.62	0.54					
**Environmental Concern**	EC1: “I would say that I am emotionally involved in environmental protection.”	0.73	0.65	0.78		0.76	0.58	0.64
	EC2: “I am worried about the worsening quality of the environment.”	0.68	0.71					
	EC3: “I think about how the environment’s quality can be improved.”	0.65	0.64				
				**Attitude**	A1: “Generally, buying recycled packaged goods is a better choice.”	0.61	0.66	0.73		0.77	0.71		0.69
A2: “In terms of price, I prefer products with recycled packaging.”	0.72	0.68										
A3: “I like shopping for products with recycled packaging.”	0.65	0.59										
A4: “Buying products with recycled packaging generally benefits consumers.”	0.67	0.83										
A5: “There’s nothing wrong with purchasing products with recycled packaging.”	0.71	0.63										
**Subjective Norms**	SN1: “Neighbours expect me to purchase products with recycled packaging.”	0.59	0.71	0.78		0.69	0.51		0.60
	SN2: “Family expects me to purchase products with recycled packaging.”	0.68	0.60					
**Perceived Behavioral Control**	PBC1: “Choosing to buy products that are in recycled packages helps to solve environmental problems.”	0.58	0.65	0.76		0.78	0.72		0.67
	PBC2: “The impact of package recycling is lower than the impacts of other measures used to mitigate the solid waste problem.”	0.79	0.60					
	PBC3: “Sorting waste and disposing of it in recycling bins is something that requires work, and it does not solve waste problems.”	0.77	0.57					
**Religiosity**	R1: “In my personal life, religion is very important.”	0.55	0.78	0.64		0.72	0.69	0.58
	R2: “Islam helps me have a better life.”	0.67	0.57					
	R3: “The Dua’aa (supplication) supports me.”	0.62	0.70					
	R4: “The Prophet Muhammad (peace-be-upon-him) is a role model for me.”	0.73	0.59					
	R5: “Performing Hajj is one of my main priorities.”	0.70	0.61					
	R6: “I believe that Allah (God) helps me.”	0.69	0.76					
	R7: “I perform all my prayers.”	0.51	0.63
	R8: “I always perform my prayers on time.”	0.65	0.83
	R9: “I perform my daily prayers in the mosque regularly.”	0.67	0.73
	R10: “I perform the obligation of Zakat.”	0.71	0.53
	R11: “I read the Qur’an regularly.”	0.61	0.69
	R12: “I fast the whole month of Ramadan.”	0.54	0.68
**Behavior**	PB1: “When shopping, I deliberately check products for environmentally harmful ingredients.”	0.67	0.78	0.70		0.79	0.56		0.61
	PB2: “When shopping, I deliberately choose products with environmentally friendly packaging.”	0.63	0.54					
	PB3: “I prefer to buy sustainable products even if they are more expensive than others.”	0.56	0.60					
	PB4: “While shopping, I look for environmental and fair trade labels before buying products.”	0.70	0.67					
**Intentions**	BI1: “I intend to buy certain products because they are less polluting.”	0.62	0.76	0.82		0.75	0.61		0.59
	BI2: “I intend to switch to other brands for ecological reasons.”	0.59	0.78			BI3: I want to buy recycled products in my coming shopping.			0.73	0.65

## Appendix

Several items were dropped due to poor factor loadings.
