# Supplementary material for: Interplay of eco-friendly factors and islamic religiosity towards recycled package products: A cross-cultural study
Source: Front Psychol. 2022 Sep 29;13:840711. doi: 10.3389/fpsyg.2022.840711 (PMC9557231; doi:10.3389/fpsyg.2022.840711)
Supplement: Supplementary file 1 [file Table_1.docx]

**Appendix**

| **Constructs** | **Items** | **Factor Loading** | | **CR** | | **AVE** | | |
| --- | --- | --- | --- | --- | --- | --- | --- | --- |
|  |  | **Pak** | **Mal** | **Pak** | **Mal** | **Pak** | | **Mal** |
| **Environmental values** | EV1: ‘‘The balance of nature is very delicate and easily upset” | 0.71 | 0.67 | 0.78 0.83 | | 0.70 0.74 | | |
|  | EV2: ‘‘Humans must live in harmony with nature in order to survive” | 0.73 | 0.75 |  |  |  |  |  |
|  | EV3: ‘‘Mankind was created to rule over the rest of nature” | 0.67 | 0.79 |  |  |  |  |  |
|  | EV4: ‘‘Humans have the right to modify the natural environment to suit their needs” | 0.82 | 0.63 |  |  |  |  |  |
| **Environmental Knowledge** | EK1. “Most smog in our big cities comes from industrial plants.” | 0.68 | 0.69 | 0.69 0.85 | | 0.72 | 0.67 | |
|  | EK2.“Unacceptable levels of mercury have been found in seafood.” | 0.78 | 0.89 |  |  |  |  |  |
|  | EK3. “Diesel fuel generates less pollution than unleaded fuel.” | 0.65 | 0.84 |  |  |  |  |  |
|  | EK4. “Ecology assumes that man is an integral part of nature.” |  |  |  |  |  |  |  |
|  | EK5. “Aluminium takes longer to decompose than iron or steel.” |  |  |  |  |  |  |  |
|  | EK6. “Traditional plastic bags do not decompose in landfills.” | 0.81 | 0.66 |  |  |  |  |  |
|  | EK7. “Products do not use any electricity in sleep mode.” | 0.69 | 0.58 |  |  |  |  |  |
|  | EK8. “Most of the water in the country is used in agriculture.” | 0.62 | 0.54 |  | |  |  |  |
| **Environmental Concern** | EC1: “I would say that I am emotionally involved in environmental protection.” | 0.73 | 0.65 | 0.78 0.76 | | 0.58 | 0.64 | |
|  | EC2: “I am worried about the worsening quality of the environment.” | 0.68 | 0.71 |  |  |  |  |  |
|  | EC3: “I think about how the environment’s quality can be improved.” | 0.65 | 0.64 |  | |  |  | |
|  |  |  |  |  | |  | | |
| **Attitude** | A1: “Generally, buying recycled packaged goods is a better choice.” | 0.61 | 0.66 | 0.73 0.77 | | 0.71 0.69 | | |
|  | A2: “In terms of price, I prefer products with recycled packaging.” | 0.72 | 0.68 |  |  |  |  |  |
|  | A3: “I like shopping for products with recycled packaging.” | 0.65 | 0.59 |  |  |  |  |  |
|  | A4: “Buying products with recycled packaging generally benefits consumers.” | 0.67 | 0.83 |  |  |  |  |  |
|  | A5: “There’s nothing wrong with purchasing products with recycled packaging.” | 0.71 | 0.63 |  |  |  |  |  |
| **Subjective Norms** | SN1: “Neighbours expect me to purchase products with recycled packaging.” | 0.59 | 0.71 | 0.78 0.69 | | 0.51 0.60 | | |
|  | SN2: “Family expects me to purchase products with recycled packaging.” | 0.68 | 0.60 |  |  |  |  |  |
| **Perceived Behavioral Control** | PBC1: “Choosing to buy products that are in recycled packages helps to solve environmental problems.” | 0.58 | 0.65 | 0.76 0.78 | | 0.72 0.67 | | |
|  | PBC2: “The impact of package recycling is lower than the impacts of other measures used to mitigate the solid waste problem.” | 0.79 | 0.60 |  |  |  |  |  |
|  | PBC3: “Sorting waste and disposing of it in recycling bins is something that requires work, and it does not solve waste problems.” | 0.77 | 0.57 |  |  |  |  |  |
| **Religiosity** | R1: “In my personal life, religion is very important.” | 0.55 | 0.78 | 0.64 0.72 | | 0.69 | 0.58 | |
|  | R2: “Islam helps me have a better life.” | 0.67 | 0.57 |  |  |  |  |  |
|  | R3: “The Dua’aa (supplication) supports me.” | 0.62 | 0.70 |  |  |  |  |  |
|  | R4: “The Prophet Muhammad (peace-be-upon-him) is a role model for me.” | 0.73 | 0.59 |  |  |  |  |  |
|  | R5: “Performing Hajj is one of my main priorities.” | 0.70 | 0.61 |  |  |  |  |  |
|  | R6: “I believe that Allah (God) helps me.” | 0.69 | 0.76 |  |  |  |  |  |
|  | R7: “I perform all my prayers.” | 0.51 | 0.63 |  | |  | | |
|  | R8: “I always perform my prayers on time.” | 0.65 | 0.83 |  |  |  |  |  |
|  | R9: “I perform my daily prayers in the mosque regularly.” | 0.67 | 0.73 |  |  |  |  |  |
|  | R10: “I perform the obligation of Zakat.” | 0.71 | 0.53 |  |  |  |  |  |
|  | R11: “I read the Qur’an regularly.” | 0.61 | 0.69 |  |  |  |  |  |
|  | R12: “I fast the whole month of Ramadan.” | 0.54 | 0.68 |  | |  | | |
| **Behavior** | PB1: “When shopping, I deliberately check products for environmentally harmful ingredients.” | 0.67 | 0.78 | 0.70 0.79 | | 0.56 0.61 | | |
|  | PB2: “When shopping, I deliberately choose products with environmentally friendly packaging.” | 0.63 | 0.54 |  |  |  |  |  |
|  | PB3: “I prefer to buy sustainable products even if they are more expensive than others.” | 0.56 | 0.60 |  |  |  |  |  |
|  | PB4: “While shopping, I look for environmental and fair trade labels before buying products.” | 0.70 | 0.67 |  |  |  |  |  |
| **Intentions** | BI1: “I intend to buy certain products because they are less polluting.” | 0.62 | 0.76 | 0.82 0.75 | | 0.61 0.59 | | |
|  | BI2: “I intend to switch to other brands for ecological reasons.” | 0.59 | 0.78 |  |  |  |  |  |
|  | BI3: I want to buy recycled products in my coming shopping. | 0.73 | 0.65 |  | |  | | |

Several items were dropped due to poor factor loadings.
